# Financial Barriers to Access to Health Services for Adult People with Disability in Iran: The Challenges for Universal Health Coverage

**Published:** 2019-03

**Authors:** Shahin SOLTANI, Amirhossein TAKIAN, Ali AKBARI SARI, Reza MAJDZADEH, Mohammad KAMALI

**Affiliations:** 1. Research Center for Environmental Determinants of Health (RCEDH), Kermanshah University of Medical Sciences, Kermanshah, Iran; 2. Department of Health Management and Economics, School of Public Health, Tehran University of Medical Sciences, Tehran, Iran; 3. Department of Global Health and Public Policy, School of Public Health, Tehran University of Medical Sciences, Tehran, Iran; 4. Health Equity Research Center (HERC), Tehran University of Medical Sciences, Tehran, Iran; 5. Department of Epidemiology and Biostatistics, School of Public Health, Tehran University of Medical Sciences, Tehran, Iran; 6. Department of Rehabilitation Management, School of Rehabilitation Sciences, Iran University of Medical Sciences, Tehran, Iran

**Keywords:** Access, Disability, Health services, Universal health coverage, Financial barriers

## Abstract

**Background:**

Reducing inequities in access to healthcare is one of the most important goals for all health systems. Financial barriers play a fundamental role here. People with disability (PWD) experience further financial barriers in access to their needed healthcare services. This study aimed to explore the causes of barriers in access to health services for PWD in Tehran, Iran.

**Methods:**

In this qualitative study, we used semi-structured in-depth interviews to collect data and selected participants through purposeful sampling with maximum variation. We conducted 56 individual interviews with people with disability, healthcare providers and policymakers from Sep 2015 until May 2016, at different locations in Tehran, Iran.

**Results:**

We identified four categories and eight subcategories of financial barriers affecting access to healthcare services among PWD. Four categories were related to health insurance (i.e. lack of insurance coverage for services like dentistry, occupational therapy and speech therapy), affordability (low income for PWD and their family), financial supports (e.g. low levels of pensions for people with disabilities) and transportation costs (high cost of transportation to reach healthcare facilities for PWD).

**Conclusion:**

Financial problems can lead to poor access to health care services. To achieve universal health coverage, government should reduce health insurance barriers and increase job opportunities and sufficient financial support for PWD.

## Introduction

Equity of access is defined as the use of healthcare conditional on the need for care (1). Reducing inequities in access to healthcare is the one the most important health policy goals for governments in any health care systems. Commitments of many governments to reduce the gap in inequities of access to health care will be failed without a clear picture of barriers of access to health care services (2). Many countries have been identifying potential barriers in access to health care (3–5), which could be different and specific for various groups of a population. Among this, people with disability (PWD) face different barriers in access to healthcare services. PWD constitute nearly 1.3% of Iran’s population (6, 7). Studies show that PWD experiences more access challenges than people without disability. For this reason, policy makers, planners and researchers need to pay more attention to the needs of PWD as one of the largest minorities in the country. In 2003, Disability Protect Act was formulated to increase access to services in Iran (8). The act aimed to provide legal protections for PWD in areas such as public building access, education, employment, inclusion and finance. Yet, 13 years passed, numerous problems such as financial and attitudinal barriers exist among PWD. Evidence is available regarding PWD’s access to healthcare services in Iran. For example, cost, transportation, cultural and environmental problems were identified as main challenges of access to rehabilitation services in Iran (9). Parents of children with intellectual disability had more negative attitude than parents of children without intellectual disability (10). Lack of awareness and financial pressures for families affected their access to speech therapy services (11). Financial factors are one of the significant barriers to access to health services in Iran. This may lead users to pay most expenses out of their pocket and face financial hardship as a result. Studies show that private share of health expenditures in Iran was higher in comparison to other middle and high income countries, while out of pocket payments as a percentage of total health expenditure consisted of 47.8% of private health expenditure for receiving health services (12), well above many other countries. This might be, we envisage, higher among PWD compared to the rest of Iranian citizens. This may challenge the goal of Universal Health Coverage (UHC), followed under the auspicious of ongoing Health Transformation Plan (HTP) that has begun since 2014 in Iran.

Despite aforementioned studies, evidence is lacking about the causes of financial obstacles for PWD’s access to healthcare services in Iran. This study aimed to explore the causes of barriers in access to health services for PWD in Tehran, the capital and most diverse and populated city of Iran.

## Methods

### Study design & data collection

We used semi-structured face to face interviews to collect data. Interviews were digitally recorded and transcribed verbatim. We provided participants with an information sheet, explained the study for them and obtained their verbal consent, while reassuring them about the anonymity and confidentiality. All in all, we conducted 56 individual interviews, each lasted about 30–90 minute and were held at different locations (interview with People with disability was held in rehabilitation centers affiliated with the Welfare Organization and the Iranian Red Crescent), from Sep 2015 until May 2016 in Tehran-Iran. SA conducted the interviews, made brief notes during data collection and documented the date, time, place, events and some important parts of conversations. Informed consent was obtained from each participant before we began the interviews.

The study was approved by the Research Ethics Committee of Tehran University of Medical Sciences (ethics code: IR.TUMS.REC.1394.1794)

### Sampling

Our samples covered three groups: people with disability and their parents, healthcare services providers (with at least one-year work experience in the context of disability) and national policymakers. We selected participants using purposeful sampling method with maximum variation to include PWD according to the type (physical and intellectual), severity of disability (mild, middle and severe), age (young and adult people), and gender (female and male). 20 people with different disabilities, 14 health service providers and 22 policymakers participated in the research. For individuals who were unable to participate in the study due to cognitive impairments, we invited their parents for participation. In addition to national relevant policy makers, we interviewed with several NGOs’ representatives as active actors in disability context. Health services providers who participated in our study had at least one year of work experience with people with disability. Interview with providers was done in their workplaces (Table 1).

**Table 1:** Characteristics of study participants

***No.***	***People with disability (PWD)***				***Healthcare providers (HSP)***			
	***Causes of disability***	***Severity of disability***	***Age***	***Male/female***	***Number***	***Profession***	***Frequency Male-female***	
1	Spinal injury	Severe	40	Female	21–22	Psychologist(P)		2
2	Cerebral palsy	Severe	35	Female	23–24	Occupational therapist (OP)	2	
3	Spinal injury	Severe	17	Male	25	Physical therapist(PT)		1
4	Spinal injury	Severe	30	Female	26	Technical Orthopedist (TO)	1	
5	Spinal injury	Severe	16	Male	27–31	Manager (M)	2	2
6	Intellectual disability	Moderate	20	Male	32	Nurse(N)		1
7	Intellectual disability	Severe	27	Male	33	Physician(PH)		1
8	Intellectual disability	Moderate	19	Male	34	Dentist(D)	1	
9	Intellectual disability	Severe	28	Male	35–36	Manufacturer(MA)	2	
10	Intellectual disability	Moderate	40	Female		Policymakers (PM)		
11	Physical	Severe	33	Female	Number	Affiliation	Male	Female
12	Multiple sclerosis	Severe	45	Female	37–40	Ministry of Health & Medical Education (MOHME)	4	
13	Multiple sclerosis	Mild	54	Male	41–45	State Welfare Organization of Iran	4	1
14	Multiple sclerosis	Moderate	38	Female	46–47	Social Security Organization	2	
15	Cerebrovascular accident	Severe	60	Male	48	Ministry of Cooperatives Labor and Social Welfare	1	
16	Physical injury	Moderate	46	Female	49	Foundation of Martyrs and Veterans Affairs	1	
17	Amputation	Moderate	57	Male	50–51	Iran Health Insurance Organization	1	1
18	Amputation	Moderate	62	Male	52	Islamic Consultative Assembly	1	
19	Physical injury	Moderate	62	Female	53–55	Ngos	2	1
20	Cerebral palsy	Mild	18	Male	56	Tehran Municipality	1	

### Data analysis

The qualitative content analysis, as described in earlier studies, using an inductive approach was applied in this study. Collecting and analysis of data were concurrent. All interviews were transcribed verbatim and was read several times for familiarization. In inductive approach, we organized qualitative data during three steps: open coding, creating categories and abstraction. All authors participated in interpretation of the coding tree. SA conducted the initial open coding through identifying and naming the phenomena that was described in the text. We first extracted meaning units from transcripts and then put a label on each meaning unit named as a code. The MAXQDA software (version 11) was a valuable tool to move codes between different parts of the data set and facilitate data analysis. After open coding, codes were grouped into seven subcategories. To reflect main message of interviews, codes were compared with each other and moved continuously across different subcategories. Finally, subcategories were sorted into three main categories. Both first and corresponding authors conducted the categorization process.

## Results

We identified four categories of financial barriers that affected access to healthcare services among PWD in Iran: health insurance, affordability, financial supports and transportation costs. Table 2 shows the financial barriers in each category.

**Table 2:** Financial barriers to access to healthcare services by PWD

***Category***	***Financial barriers and examples***
Health Insurance	Lack of insurance coverage for healthcare services such as dentistry, occupational therapy and speech therapy
	Lack of coverage for rehabilitation supplies and equipment, i.e. wheelchairs, walkers, insoles, braces, prosthetic limbs
	Disproportionate copayment for physical therapy, laboratory tests and some medicines
Affordability	Low income of people with disability and their families
Financial support	Low levels of pensions for PWD
	Mismatch between the allocation of subsides and the severity of disability and socioeconomic status
	Confusion about the condition of health costs reimbursement
Transportation costs	High cost of transport to healthcare facilities for PWD

### 1. Health insurance

We identified three subcategories in health insurance category: lack of insurance coverage for health services, lack of coverage for rehabilitation supplies and equipment and disproportionate copayment for health services such as physical therapy, laboratory tests and medicines.

#### • Lack of insurance coverage for services such as dentistry, speech therapy, and occupational therapy

During the interviews, PWD and health care providers noted the lack of health insurance coverage among PWD for some essential services, i.e. occupational therapy, technical orthopedics and speech therapy. The cost of using these services was too expensive in private settings. As a result, PWD had to abandon their treatment process:

“When, there is no insurance, especially in occupational therapy with high cost in private sector. Most families cannot pay these costs and leave the treatment and even, the effect of previous meeting is reduced, when the treatment is left” [HSP (OT), 26].

#### • Lack of insurance coverage for rehabilitation equipment and supplies such as prosthetic limbs, braces and wheelchair

Some participants complained about lack of rehabilitation supplies and equipment, i.e. wheel-chair, braces and prosthetic limbs that were not covered by current insurance schemes, which forced PWD to pay high costs to repair old equipment or buy new ones every few years, which was not affordable by many low-income families:

One service provider who was a manager at a rehabilitation center noted the experience of one of her patents to buy a pair of orthopedic shoes:

“One of the patients that had physical disorder and have to buy an orthopedic shoe. The price of a pair of orthopedic shoes was 8.000.000 Rls (equivalent to 256 USD). So, the family do not follow this issue, because they cannot afford to buy the shoe and the physical disorder might be lasting up to and also would be progressed over the time and result in additional troubles for family [HSP (M), 27].

#### • Disproportionate copayment for physical therapy, laboratory tests and medicines.

Some PWD complained about high cost and large copayment for some medical services, i.e. physiotherapy, ultrasonography and laboratory tests. Ineffective coverage of these services by the health insurance schemes, which are essential to prevent secondary conditions, decreased some low-income families’ access to such services. An example was a person with intellectual disability who needed general anesthesia for dental services, and whose parents complained about the high cost of dental services and anesthesia:

“My child used anesthesia in two steps and it cost a lot. I paid 6.000.000 Rls (equivalent to 192 USD) for each anesthesia” [PWD, 7].

### 2. Affordability

There was one subcategory entitled, “low income of people with disability and their families” in this category.

#### • Low income of people with disability and their families

Inappropriate financial status was frequently mentioned as the main barrier to PWD’s access to healthcare. This situation was more common among unemployed, low-income families, as well as families below the poverty line, which was hard for them to afford the cost of health services. These financial problems led to anxiety and depression, which jeopardized the physical and mental health of both PWDs and their family members. One participant who had child with physical disability complained about his low salary and high costs of disability.

“I am a worker and have a lot of debt. I face a lot of problems. I swear to God, I hospitalized my son in a public hospital several years ago and I have no money to discharge my son. Now, I know a needy person who has a disabled child, he works but he can’t even buy their daily necessities” [PWD, 3].

### 3. Financial Support

Three subcategories were identified here: low level of pensions for PWD, the mismatch between the allocation of subsides and the severity of disability and socioeconomic status, and confusion about the condition of health costs reimbursement.

#### • Low level of pensions for people with disabilities

The representatives of Welfare Organization mentioned the limited budget, loads of various activities, and the large number of PWD under their cover as the reasons for low-level pensions:

“Unfortunately, financial capacity of welfare organization is very low and this is because of extensive services provided by this organization. We and also disabled people are not satisfied. We estimated amount of pension about 400 to 500 thousand Rls (equivalent to 13 to 16 USD). Probably, the cost of taking a taxi to receive this money is higher” [PM (N), 42].

#### • Mismatch between the allocation of subsides and the severity of disability and socioeconomic status

The Welfare Organization subsidies the PWD to use rehabilitation services at its affiliated centers. Service providers stated that the allocated subsidies were neither in line with the severity of PWD’s disability nor their socioeconomic status. The rehabilitation centers affiliated to the Welfare Organization had limited capacity to accept PWD in line with their subsidized tariff rate. Worse still, the subsidies were not proportionally assigned in accordance with users’ socioeconomic status, so both rich and poor users enjoyed the services alike. Given the limited capacity of rehabilitation centers, this policy was against the poor’s interest to access healthcare equitably. A service provider put this in another way:

“I have an orphan disabled person in my center, whose mother suffers from cancer, while his brother is also sick and he has three other single sisters who had to leave their job because of their mother’s problem. In this condition, the subsidy is not allocated to this child in this center” [HSP (M), 28].

#### • Confusion about the condition of health costs reimbursement

Large copayments for healthcare as well as long waiting list to receive care were mentioned as barriers to access to services by some PWD. Some participants complained about the lack of clarity about the eligibility condition for reimbursements, as pointed out by a woman whose child suffered from spinal cord injury:

“I gave a receipt about 5.000.000 Rls (equivalent to 160 USD) to Welfare Organization, but at the end of the year, they said that have no budget to pay for it” [PWD, 10].

### 4. Transportation costs

We identified one subcategory: high cost of transportation, as a barrier that imposed financial tensions on PWD.

#### • High cost of transportation system for PWD

The high cost of transportation system hindered PWD to use public transport system, i.e. buses and metro, that were judged to be not easily accessible for people with physical disability. These led these people to often use Taxi, which was expensive and harder to afford than public transport:

“I have to use taxi and pay more money because I cannot cross the street” [PWD, 2].

A woman with Multiple sclerosis said:

“I have to pay at least 300 to 400 thousand Rls (equivalent to 10 to 13 USD) for taxi. I cannot use bus or metro. I have to stay at home or pay these costs” [PWD, 12].

## Discussion

### Health insurance

Our study identified the lack of health insurance as a main barrier to access to some crucial healthcare services among PWD. In particular, services that need to be used for a long time, i.e. rehabilitation services, were among those and most PWD were unable to pay. These may lead to increasing out of pocket payments and catastrophic expenditure for PWD, which in turn may result in compromising their basic needs such as food or education.

Insurance problems have been hindering access to healthcare services in other countries. Sudden changes in covered benefits and confusion about financial eligibility for Medicaid were some access barriers to healthcare services in the U.S.A. The high cost of speech therapy services was the cause of delayed utilization of such service in Iran (11). Another study showed significant relationship between the use of oral and dental health services with socioeconomic status and access to the required services in Iran (13). Oral and dental health and most rehabilitation services were not covered by health insurances and for this reason, people had to pay catastrophic proportions of their available income to access such services in Iran.

The role of insurance policies is pivotal to reduce this burden through expansion of the coverage of drug costs and lowering patients’ copayments. In addition to medicines, direct payments for rehabilitation supplies and equipment is also high in Iran. In the current study, PWD mentioned that they had to use low-quality wheelchairs and braces as a result of ineffective health insurances. Further, lack of access to affordable assistive technologies (AT) can have negative effects on health status and community participation and inclusion of PWD (14). Government should create infrastructures and financial resources to produce, purchase and allocate AT for different types of disabilities. Reinforcement of government leadership and governance in production of domestic and low-cost AT on a big scale, plus designing public funding schemes are two essential measures to increase PWD’s access to AT.

The cost of such public services in rehabilitation centers affiliated to the Iranian Red Crescent was considerably lower than the private sector, so even the poor could receive services free of charge. It would be beneficial if the government could expand these activities by formulating supportive and motivating policies such as allocating subsidies and donors’ financial assistance towards PWD and through separate channels.

As the most comprehensive concept ever advocated by the WHO, universal health coverage (UHC) requires provision of quality services for all population without facing any financial hardship (15). UHC would not be achieved unless all citizens, i.e. PWD enjoy the healthcare services that they need, including promotion, prevention, treatment, rehabilitation, and palliative care, without financial difficulties. Effective health insurance for all people is therefore essential to reach UHC. Unless the public share of health expenditure increases substantially for services used by PWD, UHC cannot be achieved in Iran. Policymakers should more focus on health needs of disadvantaged groups like PWD and formulate policies to increase financial risk protection for PWD who use healthcare services.

### Affordability

Our study identified most PWD participants either as jobless or low-paid workers, which may lead to poverty and lower financial access to health care among PWD. Physical and environmental barriers, i.e. inappropriate streets, sidewalks and buildings were the most important barriers to PWD’s employment in Iran (16, 17). Further, these factors can lead to more job stresses among PWD (16). Poverty and disability have complicated and multidirectional relationship (18). Disability can increase health needs, the unemployment rate and the risk of poverty among PWD. The government needs to implement more motivating polices, i.e. loans, tax discount schemes for employers to encourage them hire PWD, as well as allocate more pension and cash subsidy to persons who are more likely to face catastrophic financial problems, i.e. persons with severe and profound disabilities. Moreover, the municipalities need to develop their knowledge and monitoring capacities to build public spaces with appropriate access to public transport and spaces, aiming to improve social inclusion of PWD.

### Financial supports

Most PWD who participated in our study noted that financial support of the Welfare Organization, e.g. pension or reimbursement was neither sufficient nor adjusted for the severity of disability and their socioeconomic status. In Iran, insufficient financial support is the main reason for not using speech therapy services by PWD (11). Worse still, inadequate coverage of some inpatient services (e.g. rehabilitation services and dentistry), medicines, equipment and supplies, as well as low pensions for PWD may lead to poorer access to healthcare services among PWD. Some inpatient services, e.g. dentistry services can encounter PWD with catastrophic costs. Thus, policymakers need to address the problem immediately. PWD need suitable job and income to participate actively in the society (19). Hence, for those persons who are not able to become employed, government should consider sufficient pension, especially people who are the head of a household, to enable them to fulfil their needs, i.e. health and education. Increasing financial supports to PWD and reducing their financial hardship to access to healthcare needs collaborative attempts of all public policymakers, i.e. the MoHME (20). New and more sustainable resources, i.e. sin and luxury taxes from tobacco and soft drinks can be collected and used towards such targets.

## Conclusion

Inadequate coverage of some healthcare services, medicines, equipment and supplies have all contributed to spiraling health expenditures among PWD in Iran. Worse still, unemployment worsens low income and poorer access to healthcare services among PWD. To achieve universal health coverage (UHC) in Iran, reducing barriers to effective health insurance for PWD to ensure their equitable access to needed healthcare services is crucial. Unless greater job opportunities will be created for PWD and an effective model will be in place to distribute and allocate sustainable and sufficient financial support for those who are unable to work, UHC would be challenging to achieve in Iran and perhaps anywhere. Governments, as stewards of the health system are therefore responsible to create fruitful and fair societies to ensure all conditions are in place for all citizens including PWD, to fulfill all dimensions of UHC.

## Ethical considerations

We fully observed the ethical considerations. The study was approved by the Research Ethics Committee of Tehran University of Medical Sciences (ethics code: IR.TUMS.REC.1394.1794).
